# The Forceps

**Published:** 1869-08

**Authors:** 


### The Forceps.

In the use of these instruments there has been a remarkable reaction from the overcautious practice which prevailed towards the end of the last century, and which was due in a great measure to the interference and example of the illustrious Denman, who taught accoucheurs to place an almost Abyssinian confidence in the powers of nature, and never to resort to instruments unless they had the most positive evidence that she was unequal to her task. He went so far as to insist that the head of the child should have rested for six hours as low as the perineum, *i. e.* in the situation which will best allow of their application, before forceps should be applied, although the pains should have altogether ceased for that time.

In Denman's time there were no examinations in midwifery, and the science of obstetrics was slighted and despised by the medical colleges; but now when medical men are so much better instructed in midwifery, the forceps are much more frequently resorted to, and with the very best results. The statistics of the Dublin Lying-in-Hospital prove that the maternal deaths have steadily decreased as the forceps have been more frequently and earlier used. Latterly they have been brought in play 232 times in 6053 cases, or about once in 26; Carus, of Dresden, uses them as often as once in 14 cases; Busch of Berlin once in 12; Siebold, of Berlin, once in 7.

Swayne now makes it a rule to apply the forceps wherever the head has remained immovable in the pelvic capacity for more than two hours. But if the head advances ever so slowly, while the pulse continues good, the abdomen remains free from pressure, and there is no obstruction to the emission of the urine, then the forceps should not be used until the child is dead.

Swayne has applied the forceps ninety times; of these cases, 69, or more than three-fourths, were in primiparæ. This might be expected, for in forceps cases there is generally both increased resistance and diminished uterine power; both of which are apt to occur in first labors, especially if the woman be rather old. Of Swayne's 69 cases in primiparæ, 10 were 38 to 43 years old.

In 41 cases out of the 90, there was want of power with slight want of room in the pelvis.

In 22 cases out of the 90 there was want of power only. In 21 cases there was want of room only.

Convulsions occurred in four cases; and prolapse of the cord in two.

Four cases died in which the forceps were used, viz., two from puerperal fever, one from bronchitis, and another from malignant scarlet fever.

Swayne prefers the short forceps of Simpson, although he uses the long kind occasionally.—*British Medical Journal.—Medical Gazette.*

